# Computed Tomography-based Analysis of Tibial Tuberosity–Trochlear Groove Distance in Indian Population

**DOI:** 10.7759/cureus.5277

**Published:** 2019-07-30

**Authors:** Balgovind S Raja, Hariharan Mohan, Akash M Jain, Sai Gautham Balasubramanian

**Affiliations:** 1 Orthopaedics, K. E. M. Hospital & Seth G. S. Medical College, Mumbai, IND; 2 Orthopaedics, Sir H. N. Reliance Foundation Hospital and Research Institute, Mumbai, IND; 3 Orthopaedics, K. E. M. Hospital and Seth G. S. Medical College, Mumbai, IND

**Keywords:** knee, tt-tg distance, knee anthropometry

## Abstract

Aim

To study the tibial tuberosity-trochlear groove distance (TT-TG) in normal Indian population and the variation of the same in relation to tibial size using computed tomography (CT) of knee.

Methods

CT of 100 knees (62 males and 38 females) were assessed. TT-TG distance and maximal medio-lateral (MML) distance of tibia was measured on axial CT scans. The modified TT-TG (mTT-TG) was calculated as the ratio of TT-TG and MML.

Results

The average TT-TG distance was 13.01 (±2.84) mm for the entire group with males and females having 12.82 (±2.95) and 13.32 (±2.66) mm, respectively (p > 0.05). The MML distance was 75.99 (±3.78) and 66.77 (±4.33) mm for males and females, respectively (p < 0.05). The average modified TT-TG was 0.18 ± 0.04. The TT-TG distance of Indian knees was similar to values obtained in Caucasian knees and higher than other Asian knees (p < 0.05).

Conclusion

The average TT-TG distance in Indian population is 13.01 mm, with no difference between males and females. The ML/TT-TG ratio was 0.18. The TT-TG distance in Indian population is found to be similar to the Western population and significantly higher than other Asian population.

## Introduction

Tibial tuberosity-trochlear groove distance (TT-TG) relates to the relationship of the tibial tuberosity with the femoral trochlea and is used to assess the degree of external torsion and lateralisation of the tibial tuberosity in patellar instability [1]. Understanding of the TT-TG distance provides a valuable edge in distal realignment knee procedures. Ethnical variations in knee morphometry or dimensions have been reported in literature and the Indian knees are found to be different in comparison to the Caucasians [2]. Of the present literature concerning to the TT-TG distance, there is a lack of clarity of the same in Indian population. Kulkarni et al. using magnetic resonance imaging (MRI) studied the relation of tibial tuberosity in relation to femoral trochlea [3]. But MRI-based studies are seen to underestimate the TT-TG distance. MRIs performed on dedicated knee coils are inaccurate for the measurement of tibial tuberosity trochlear groove distance owing to the partial flexed position of knee in the console [4]. Computed tomography (CT) is the gold standard investigation for assessing TT-TG distance [1,5].

We here present a CT-based assessment of TT-TG distance in normal Indian population and the variation of the same in relation to tibial size. The null hypothesis kept at the start of the study was that TT-TG distance in Indian population was different from Western population.

## Materials and methods

This was a CT scan-based observational study of 100 knees. CT scans of patients who had undergone CT angiography of lower limbs for various reasons with normal knee CT, were used for the study. The sample size of 100 knees was calculated on the basis of study by Mullaji et al. [6]. CT scans showing malalignment, fracture, previous knee surgery and arthritic changes were excluded from the study.

**Figure 1:**
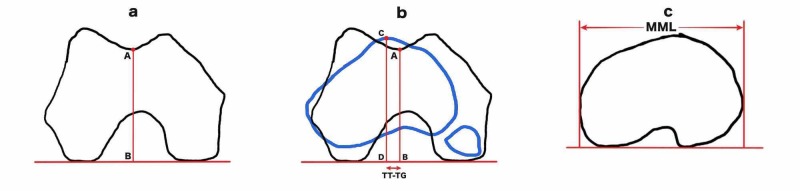
Illustrations showing measurement of TT-TG distance and MML distance. a) Point A marked in the deepest point of trochlear groove. Line AB drawn perpendicular to posterior condylar line. b) Distal femur cut superimposed on proximal tibia. Point C marked in the anterior most point of tibial tuberosity. Line CD drawn perpendicular to posterior condylar line. Distance between the lines AB and CD is measured as TT-TG distance. c) Proximal tibial cut showing measurement of MML. TT-TG: Tibial tuberosity-trochlear groove; MML: Maximal medio-lateral.

The TT-TG distance was measured between the most anterior point of tibial tuberosity with the deepest bony point of trochlear groove which is perpendicular to the tangent to bony borders of the posterior condyles of distal femur on axial CT scans [5]. First, a line was drawn on the axial slice with Roman arch best seen tangential to the posterior femoral condyles (posterior condylar reference line) and to this a perpendicular line was drawn through the deepest point of the trochlear groove (Figure 1a). These lines were then transferred to the axial section with the most anterior point of the tibial tuberosity. A perpendicular line was drawn to posterior condylar reference line through the most anterior point of the tibial tuberosity. The distance between these two perpendicular lines constituted bony TT-TG distance and was measured in millimetres (Figure 1b). The maximal medio-lateral (MML) axis was measured in the tibial platform slice where the posterior condylar notch was clearly evident. MML was defined as distance between two lines (medial and lateral) perpendicular to the posterior condylar line of tibia (Figure 1c). The modified TT-TG (mTT-TG) was calculated as the ratio of TT-TG and MML (TT-TG/MML) [7].

Measurements were made using Horos Dicom viewer. The data obtained was assessed using Microsoft Excel 2010 and XL-STAT software. The values were expressed as mean ± standard deviation. The normality of test was assessed with Shapiro-Wilk test. Student’s t test or Wilcoxon-Mann-Whitney test was used to assess the statistical significance and p-value of <0.05 was considered significant. The normal range of MML-TT-TG was determined with 95% confidence interval. The TT-TG values obtained in our study were compared with similar studies in literature on Caucasian and other Asian population [1,8,9]. T-test comparing the mean of two groups was used for comparison.

## Results

The mean TT-TG distance for the entire study group was 13.01 mm (±2.84). There was no significant difference in TT-TG distance between male and female population (p > 0.05). The MML distance was 75.99 mm (±3.78) and 66.77 mm (±4.33) for the male and female group, respectively. This was found to be statistically significant (p < 0.05). The average ML/TT-TG ratio (mTT-TG) was 0.18 (±0.04). There was significant difference in this ratio between male and female subjects. The ML/TT-TG ratio averaged 0.18 (±0.04) with males and females having 0.17 (±0.04) and 0.2 (±0.05), respectively. This difference was found to be statistically significant (p < 0.05) (Table 1).

**Table 1:** Patient demographics and study results. TT-TG: Tibial tuberosity-trochlear groove; MML: Maximal mediolateral distance; mTT-TG: Modified tibial tuberosity-trochlear groove.

	Male	Female	Total	P value
Number of subjects	62	38	100	
Mean age in years	39	44	41	
TT-TG				
Mean	12.82	13.32	13.01	0.395
Standard deviation	2.95	2.66	2.84	
MML				
Mean	75.99	66.77	72.48	<0.0001
Standard deviation	3.78	4.33	6	
mTT-TG				
Mean	0.17	0.2	0.18	0.001
Standard deviation	0.04	0.05	0.04	

The mean TT-TG distance of our study was significantly higher compared to other Asian population. Song et al. reported a mean TT-TG distance of 10.24 (±0.8) mm for Korean population [9]. There was no significant difference between our values and those obtained for Caucasian population (Table 2).

**Table 2:** Comparison of our results with similar studies in literature. TT-TG: Tibial tuberosity-trochlear groove

	Our study	Alemparte et al. [8]	Song et al. [9]	Dejour et al. [1]
Place of study	India	Chile	Korea	France
Method	CT	CT	CT	CT
Number of knees	100	60	100	27
TT-TG Mean	13.01	13.6	10.24	12.7
TT-TG Standard deviation	2.84	8.8	0.8	3.4
P value	-	0.5362	<0.0001	0.6306

## Discussion

Patellar instability is a relatively common orthopaedic problem for which the TT-TG distance is a predominant factor in evaluation. An increased tibial tuberosity-trochlear groove distance is considered as an isolated and independent predisposing factor leading to patellofemoral instability and even more as an indication for performing distal realignment procedure around knee like medialisation of the tibial tuberosity [10]. It is one of the gold standard diagnostic measurements which may affect about 56% of patients with patellar dislocation [1,10,11].

The tibial tuberosity position relative to the femoral trochlea is pertinent for the quadriceps mechanism to function normally as it decides the direction of the inferolateral force vector on the patella and pull of the quadriceps mechanism [3]. In a normal individual the tibial tuberosity is placed more in line under the sulcus of femur, so the inferior force vector is much higher in magnitude than the lateral force vector. This aids in preventing the lateral subluxation of patella. But, if the tibial tuberosity is located more laterally the lateral force vector becomes higher in magnitude which eventually tries to subluxate or dislocate patella laterally [3]. An increased tibial torsion hence leads to patellar maltracking which is characterised by alteration in quadriceps vector and is radiologically assessed with TT-TG distance.

A TT-TG distance in the range 10-15 mm is usually considered as normal, and an increased distance is an indication for surgical intervention in the form of medial tibial tuberosity transfer to correct patellofemoral malalignment [12]. However, the exact upper limit of TT-TG distance above which surgical intervention is needed is still a topic of debate and needs further detailed evaluation. Dejour et al. considered a distance of 20 mm or more to be pathologic in symptomatic patients, while Koëter et al. stated that medialisation of the tibial tubercle to be done when a threshold of 15 mm was exceeded [1,13]. This lack of uniformity may be attributed to the differences in the sizes of knee joints of patients with patellofemoral instability and hence the same TT-TG distance would have different clinical significance for different knee joints. Studies have shown that a TT-TG distance of even <15 mm may predispose to patellofemoral malalignment [14,15]. Therefore, a cut-off of 20 or 15 mm for all patients may not be appropriate, instead individual morphological factors must be considered while measuring the TT-TG distance.

Our study revealed the mean TT-TG distance to be 13.01 (±2.84) mm with no significant difference between gender. Twenty-four percent of the subjects had a value of more than 15 mm. Dejour et al. used CT scans with improved accuracy and reliability to calculate the TT-TG distance [1]. They recorded a TT-TG distance of 12.7 (±3.4) mm in 27 knees. Alemparte et al. in about 60 knees using CT found an average value of 13.6 (±8.8) mm as TT-TG distance with males having significantly larger vales than females [8]. Though literature exists regarding the relation of tibial tuberosity with femoral trochlea, these studies were predominantly done in Caucasian population. Only a single study by Kulkarni et al. exists with respect to Indian population [3]. The above-mentioned study was MRI-based with a sample size of 100 but, the authors utilised the distal insertion of patellar tendon as the reference for tibial tuberosity. In effect they measured the patellar tendon-trochlear groove (PT-TG) distance which is found to be different from the original TT-TG distance. Moreover, MRI-based studies done in dedicated knee coils are seen to have significantly different TT-TG distance compared to an MR body coil [4].

Pennock et al. found an association of TT-TG distance with height and observed a variation of TT-TG distance by 0.12 with each centimetre increment [16]. Balcarek et al. witnessed no relation of the TT-TG distance with patient age or femur width, but still proposed the TT-TG distance to be interpreted based on knee size [17]. In an attempt to seek a more reliable and standardised method to measure TT-TG distance, Cao et al. modified the accepted method of measurement and used modified TT-TG (mTT-TG) which is the ratio between TT-TG and tibial MML axis [7]. They recorded a range of 0.11 to 0.25 as normal and proposed a value of >0.25 associated with patellofemoral malalignment [7]. The ML/TT-TG ratio in Indian population was found out to be 0.18 (±0.04). Males were found to have a larger MML compared to females (75.99 ± 3.78 vs 66.77 ± 4.33) and in turn the mTT-TG ratio is significantly larger in females in comparison to males (0.20 ± 0.05 vs 0.17 ± 0.04) with a P-value < 0.05.

The study has its limitations. Firstly, the sample size we had in our study was small. But, this is comparable to the rest of the literature at present. Secondly, MRI-based assessment of the TT-TG distance is being considered by many as a single investigation that would identify the underlying pathology and assess the status of the cartilage. But, there is a lack of clarity in respect to the validity of the techniques as Aarvold et al. have demonstrated that the MRI done with dedicated knee coils often gives altered distance [4]. This study to our knowledge is the first in Indian population to calculate the TT-TG distance using the ‘gold standard’ computed tomography and will aid in providing references for the future studies and a benchmark for distal knee realignment procedures.

## Conclusions

The present study reveals the average TT-TG distance to be 13.01 mm in Indian population with males having average distance of 12.82 mm and females 13.32 mm. The ML/TT-TG ratio was 0.18. The TT-TG distance in Indian population is found to be similar to the Western population and significantly higher than other Asian population.
